# Dynamic analysis of the mesenchymal-epithelial transition of blood-brain barrier forming glia in *Drosophila*

**DOI:** 10.1242/bio.020669

**Published:** 2017-01-20

**Authors:** Tina Schwabe, Xiaoling Li, Ulrike Gaul

**Affiliations:** 1 Department of Biochemistry, Gene Center, Center of Integrated Protein Science (CIPSM), University of Munich, Feodor-Lynen-Str. 25, Munich 81377, Germany; 2 Rockefeller University, 1230 York Ave, New York, 10065-6399 NY, USA

**Keywords:** MET, Blood-brain barrier, GPCR signaling, Epithelial morphogenesis, *Drosophila*

## Abstract

During development, many epithelia are formed by a mesenchymal-epithelial transition (MET). Here, we examine the major stages and underlying mechanisms of MET during blood-brain barrier formation in *Drosophila*. We show that contact with the basal lamina is essential for the growth of the barrier-forming subperineurial glia (SPG). Septate junctions (SJs), which provide insulation of the paracellular space, are not required for MET, but are necessary for the establishment of polarized SPG membrane compartments. *In vivo* time-lapse imaging reveals that the Moody GPCR signaling pathway regulates SPG cell growth and shape, with different levels of signaling causing distinct phenotypes. Timely, well-coordinated SPG growth is essential for the uniform insertion of SJs and thus the insulating function of the barrier. To our knowledge, this is the first dynamic *in vivo* analysis of all stages in the formation of a secondary epithelium, and of the key role trimeric G protein signaling plays in this important morphogenetic process.

## Abstract (precis)

**Summary:** This study examines the major steps and underlying mechanisms of mesenchymal-epithelial transition of the blood-brain-barrier forming glia in *Drosophila*, including the role of basal lamina, septate junctions and of trimeric G protein signaling.

## INTRODUCTION

By forming a selective diffusion barrier, epithelia protect the body from the environment and promote the establishment of different chemical milieus within it. Understanding the mechanisms that drive the cellular rearrangements necessary for the formation of epithelial sheets is thus fundamental to our understanding of the development and evolution of multicellular organisms.

Based on their mode of formation we distinguish primary epithelia, which arise by shape changes of the original blastoderm epithelium, and secondary epithelia, which form from mesenchymal intermediates by a process called mesenchymal-epithelial transition (MET). MET is crucial for the development of many tissues and organs, such as kidney tubules, the blood vascular system, the heart, the embryonic trophectoderm and the somites in vertebrates, as well as the heart, midgut, follicle cells and blood-brain barrier (BBB) in *Drosophila* (Barasch, 2001; Tepass, 2002; Tepass and Hartenstein, 1994). Secondary epithelia have in common the lack of an adherens junction belt, and instead form spot adherens junctions; they also lack the classical apical-basal organization, as characterized by the apical Crumbs complex, Bazooka, together with the cadherin-catenin complex at the adherens junction and lateral/basal complex with Lethal giant larvae (Tepass, 2012). Instead, they establish apical-basal polarity by other means, which we are examining in this study. The MET is the converse of the epithelial-mesenchymal transition (EMT), which is very well studied due to its relevance for tumor metastasis (Baum et al., 2008; Serrano-Gomez et al., 2016; Seton-Rogers, 2016; Ye and Weinberg, 2015; Zhang et al., 2016). In contrast, MET has received less attention (Chaffer et al., 2007; Combes et al., 2015; Takahashi et al., 2005; Trueb et al., 2013), and thus our understanding of the morphogenesis of secondary epithelia remains sketchy. To form an epithelium, mesenchymal cells need to switch from a motile to a stationary state and align their polarity with that of their future neighbors. In doing so, cells need to upregulate expression of epithelium-specific genes, such as E-cadherin, while down-regulating expression of mesenchyme-specific genes (Barasch, 2001). Finally, cells must coalesce and form cell-cell junctions in a highly coordinated manner in order to create a regularly patterned epithelium (Barasch, 2001; Nelson, 2009; Schmidt-Ott et al., 2006).

Studies on the development of kidney tubules in vertebrates, as well as the heart and midgut in *Drosophila*, demonstrated that contact to neighboring tissues is essential to transform mesenchymal into epithelial cells, while interactions with proteins of the extracellular matrix (ECM) are thought to be necessary for the establishment of polarity (Hollinger et al., 2003; Rodriguez-Boulan and Nelson, 1989; Tepass and Hartenstein, 1994; Yarnitzky and Volk, 1995). Molecules regulating MET include transcription factors, signaling pathways, such as FGF receptor, BMP and Notch pathways, integrins, cadherins, claudins and Rho GTPases (Boyle et al., 2011; Jülich et al., 2005; Khairallah et al., 2014; Li et al., 2014; Lindstrom et al., 2015; Nakaya et al., 2004; Sanchez et al., 2006). In the current study, we describe trimeric G protein signaling as an important pathway that coordinates cell growth during secondary epithelium formation.

**Fig. 1.**
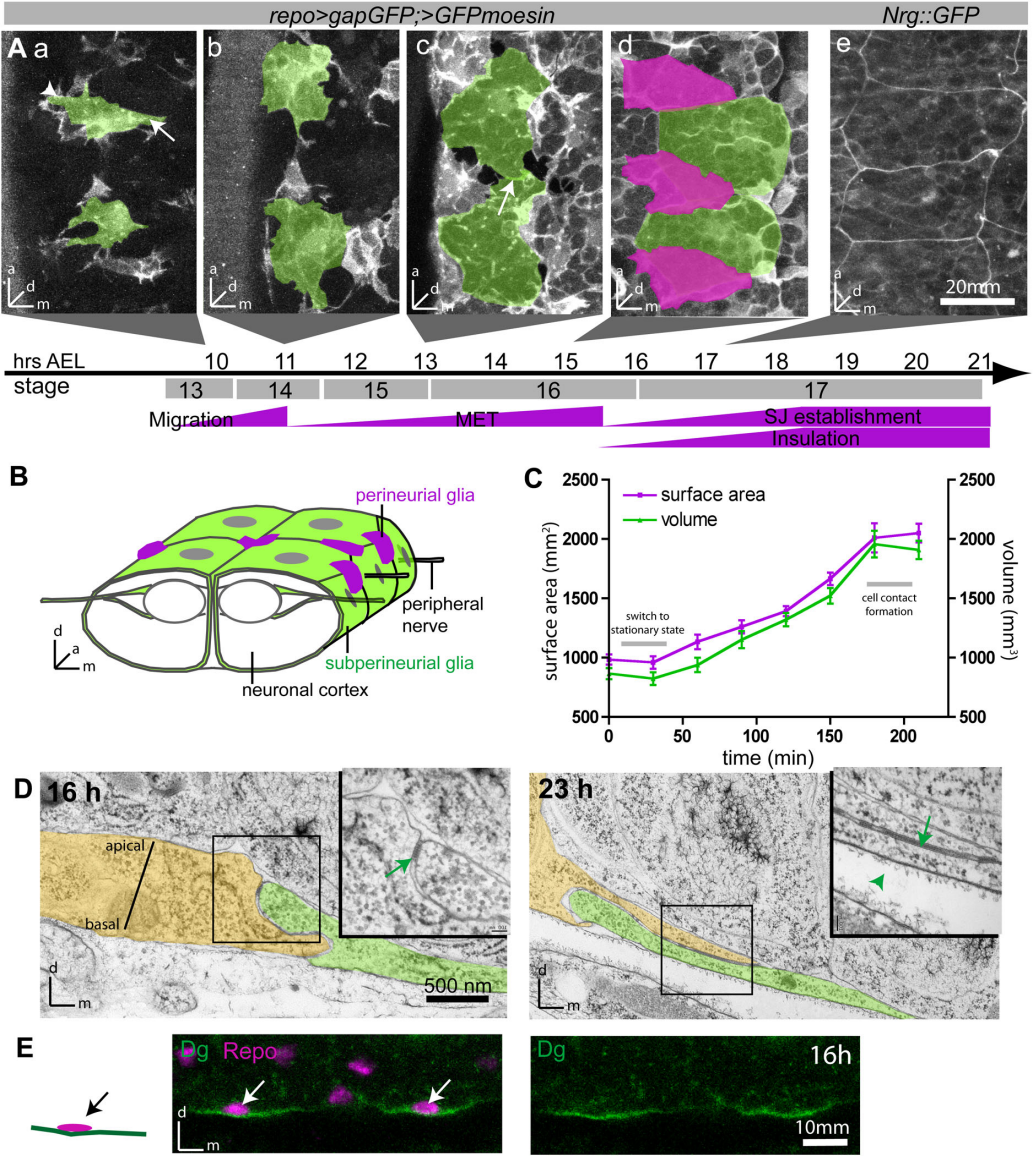
**Development of the BBB during embryogenesis.** (A) Timeline of glial MET in live embryos. (a) SPG migrate to the surface of the nerve cord and display a broad leading (arrowhead) and a narrow trailing edge (arrow). (b-d) SPG grow until they cover the entire CNS surface and contact their neighbors (arrow in c). (e) Subsequently, SJ material accumulates along regions of cell contact. (a-d) Glia are labeled by *repo>GFPmoesin* and >*gapGFP* and imaged live; SPG highlighted in green; perineurial glia (PNG) in magenta in (d). (e) SJs labeled with Nrg::GFP. Ventral views of the CNS surface, midline to the right; 5-10 µm confocal stacks. Bottom panel indicates age of embryos raised at 25°C. (B) Schematic organization of the SPG epithelium (green) ensheathing the ventral nerve chord. (C) Time course of SPG growth between 10.5 and 13.5 h. Shown are mean±s.e.m., *n*=12. (D) Electron microscopy images of 16 and 23 h embryos showing two SPG cells in green/yellow and overlying basal lamina (arrowhead in inset). Accumulation of SJs (arrows) is accompanied by increasing cell-cell overlap. (E) Dystroglycan (Dg; green) localizes to the basal side of SPGs (nuclei labeled in magenta with Repo). Orthogonal view, CNS facing up; ventral SPG nuclei indicated by arrows.

The CNS of *Drosophila* is protected by a blood-brain barrier (BBB), which is required for the maintenance of ionic homeostasis within the CNS by shielding neurons from high concentrations of potassium and glutamate in the surrounding hemolymph. In addition, the barrier selectively regulates the uptake of nutrients from and the release of waste products to the hemolymph. The barrier is established by subperineurial glial cells (SPG), which form a squamous, secondary epithelium that envelops the CNS as a whole (Fig. 1B). Similarly to other secondary epithelia, such as the heart and midgut (Medioni et al., 2008; Tepass, 1997), SPG do not form a contiguous adherens junction belt, but spot adherens junctions (Schwabe et al., 2005). The insulation of the paracellular space is achieved by the establishment of long septate junction (SJ) belts along glial cell contacts at the lateral membrane. The ultrastructure and composition of these SJs are comparable to those of primary epithelia (Baumgartner et al., 1996; Fehon et al., 1994; Hijazi et al., 2011; Syed et al., 2011). SJs form an array composed of individual septa spanning the paracellular space (Fig. S1). Tracer studies have shown that individual septa act as impartial filters, and it is thought that the number of aligned septa determines the tightness of the paracellular barrier (Abbott, 1991).

The *Drosophila* BBB is an interesting model to gain insight into the mechanisms of MET, as it forms relatively rapidly during embryonic development (Schwabe et al., 2005), and its physiological function is easy to probe experimentally by measuring the diffusion of various tracers into the CNS. At present, it is still unknown how SPG transition from a migratory mesenchymal to a stationary, epithelial state, and a few components involved in BBB formation, have been identified. Among those is a G protein coupled receptor (GPCR) signaling pathway, which consists of the orphan GPCR Moody, the regulator of G protein signaling (RGS) Loco, as well as two heterotrimeric G proteins (Gαi-βγ, Gαo-βγ). Both under- and overactivity of the pathway result in BBB insulation defects (Granderath et al., 1999; Schwabe et al., 2005). Cell biological analysis showed that these defects are caused by a maldistribution and shortening of the insulating glial-glial SJs (Schwabe et al., 2005). However, it remains unclear which aspects of BBB formation are regulated by the pathway and by which mechanism the SJ distribution is ultimately affected.

Here we present a detailed cell biological analysis of the major stages of BBB formation, namely SPG migration, polarity establishment, cell growth, cell contact and SJ formation. We find that SJs, apart from their role in insulation, act as a fence that is essential for establishing distinct membrane compartments within SPG. Glial growth and epithelial closure, in turn, require adhesion to the basal lamina and are modulated by Moody pathway activity. *In vivo* time-lapse imaging reveals that G protein signaling regulates SPG growth and cell shape by controlling protrusive activity and stability at the leading edge. Strikingly, over- and underactivity of the Moody pathway show distinct subcellular phenotypes during epithelium formation, although the ultimate result, a leaky BBB, is the same in both cases.

## RESULTS

### Time course of SPG forming a secondary epithelium

To analyze the dynamics of SPG behavior as they undergo MET, we performed time-lapse imaging. As SPG are very thin, we used a combination of two fluorescent markers (gapGFP and moesinGFP), driven by repo-Gal4, to robustly visualize their shapes. The MET process occurs quite rapidly during embryogenesis, from about 9 to 19 h after egg laying (AEL) at 25°C (equivalent to Hartenstein stages 13-17; Campos-Ortega and Hartenstein, 1985). Between 9 and 11 h, individual SPG migrate to the CNS surface. During their migration, the cells show a clearly polarized morphology, with a broad leading and a narrow trailing edge (Fig. 1Aa) (Ito et al., 1995; Schmidt et al., 1997). They then become stationary and grow extensively in a lateral direction to eventually form a contiguous sheath that is composed of relatively few large cells and envelops the CNS as a whole (Fig. 1A-C; Movie 1). Remarkably, the growth of the SPG is both synchronous and isometric, such that all cells have a compact shape and are of similar size as their neighbors at any given time.

By 13 h, the SPG cover most of the CNS and begin to contact their neighbors (Fig. 1Ac). Epithelial closure is largely completed between 14.5 and 15.5 h (Fig. 1Ad). We define epithelial closure as cells establishing continuous cell contacts across their lateral membranes without visible gaps between them. Subsequently, the barrier forming SJs accumulate at the lateral membrane compartment, as visualized by an endogenous fusion of the SJ component neuroglian (Nrg) to GFP (Nrg::GFP; Fig. 1Ae), a faithful marker for SJ formation (Schwabe et al., 2005). Neighboring SPG form extensive membrane overlaps, thereby increasing the width of the lateral membrane compartment (Schwabe et al., 2005), and our ultrastructural analysis shows that SJ material accumulates as the membrane overlap increases (Fig. 1D), suggesting that the two processes are connected.

**Fig. 2.**
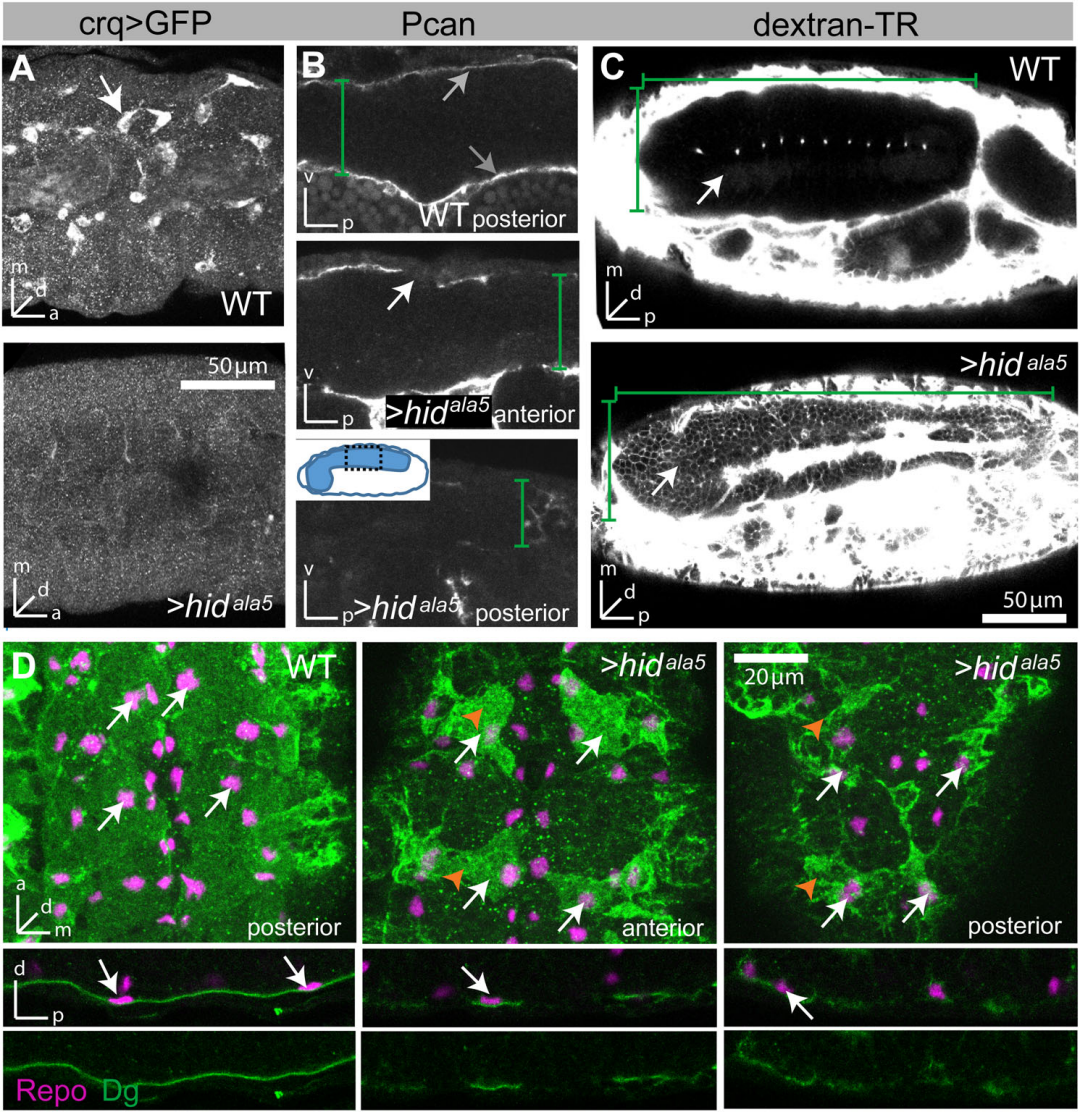
**The basal lamina is required for SPG growth.** (A) *UAS-hid^ala5^* driven by *crqGal4* efficiently ablates embryonic hemocytes (labeled with UAS-GFP). Stage 16 embryos; lateral view, 20 µm stacks. (B) In *crq>hid^ala5^* embryos, Perlecan (Pcan) staining (gray arrows) is strongly reduced, indicating depletion of the basal lamina or loss of basal lamina integrity. Depletion is more severe in the posterior nerve cord. Lateral views at stage 16. White arrow indicates loss of ECM. (C) In WT embryos, a 10 kD dextran-TexasRed dye is completely excluded from the CNS (outlined by green brackets); only the midline channels appear as a single line of fluorescent spots. In contrast, in *crq>hid^ala5^* embryos the BBB does not form, and the dye freely penetrates into the CNS (arrow). Note that due to the near-loss of basal lamina integrity, the nerve chord does not condense. In B and C green brackets delineate dimensions of nerve cord. (D) In *crq>hid^ala5^* embryos, SPG (orange arrowheads) show severe growth defects in an anterior-to-posterior gradient, but asymmetric localization of Dystroglycan (Dg) is maintained. SPG are labeled with Dg (green) and all glia nuclei with Repo (magenta, white arrows). Since the nerve chord has not condensed, fewer cells are visible in the same section when comparing WT with mutant. Stage 16; upper panels are ventral views; stacks of 10-13 µm. Lower panels are cross section views of the CNS; single sections.

Finally, as SJs accumulate, insulation of the paracellular space improves rapidly, as shown by exclusion of a hydrophilic dye from the nervous system from 18.5 h onwards (Fig. 2C) (Schwabe et al., 2005), indicating that a functional BBB has been established.

Accessory cells often play an important role during the development and function of secondary epithelia, such as improving mechanical stability (Rugendorff et al., 1994; Tepass and Hartenstein, 1994). We and others have identified a second, distinct type of glia located at the CNS surface, named perineurial glia (PNG) (Fig. 1Aa; Fig. S1) (Ito et al., 1995; Stork et al., 2008). In the embryo, we define PNG as individual squamous cells that are located between the basal lamina and the SPG epithelium (Fig. S1). Repo-Gal4 drives expression in both SPG and PNG, but the two glial types are easily distinguished by location around the nerve chord and by morphology (Fig. S1; Fig. 1Ad). While PNG and SPG appear at the same time on the ventral nerve chord (VNC) surface, PNG nuclei are in different XY locations to SPG nuclei. PNG cells assume a triangular shape and are actin-rich, thus appearing brighter in our assay due to higher levels of moesin-GFP labeling, whereas the SPG assume a rectangular shape, contain less actin and therefore appear less bright (Fig. S1).

The lack of an early PNG-specific driver precluded an analysis of the specific function of the PNG during epithelium formation. However, our time-lapse images reveal frequent filopodial contacts between SPG and PNG, as well as stereotyped PNG positioning relative to the SPG, suggesting that PNG might serve as guideposts (Movie 1). During SPG epithelium formation PNG neither integrate into the SPG epithelium nor form a separate epithelium, but rather remain individual cells that sit atop the SPG, facing the basal lamina. They proliferate during larval growth to form a layer of cells located between the basal lamina and the SPG (Fig. S1) (Stork et al., 2008).

### SPG growth and polarization require basal lamina and SJ belt

We next sought to investigate the molecular mechanisms that regulate the various aspects of the SPG MET. *In vitro* studies have shown that adhesion to extracellular matrix (ECM) components is both necessary and sufficient to promote the (non-proliferative) growth and polarization of cells (Huang and Ingber, 1999). Contact with the ECM is similarly required for glial wrapping of the peripheral nerves (Xie and Auld, 2011). The SPG are in direct contact with a basal lamina, which is secreted by hemocytes and surrounds the developing nervous system (Fig. 1D; Fig. S1) (Evans et al., 2010; Martinek et al., 2008; Olofsson and Page, 2005; Tepass and Hartenstein, 1994). These hemocytes originate from the head mesoderm and migrate posteriorly along well-defined routes (Cho et al., 2002). We find that SPG express the laminin and perlecan receptor dystroglycan (Dg) (Schneider et al., 2006). Even prior to epithelial closure, Dg specifically localizes to the side of the SPG that faces the basal lamina, i.e. the nervous system-distal side (Fig. 1E). Thus, our data suggest that SPG form contacts with the basal lamina and that this contact results in a first apical-basal polarization of the cells.

To directly test the role of the basal lamina for SPG growth, we ablated embryonic hemocytes by specifically expressing a constitutively active form of the pro-apoptotic factor Hid (*crq>hid^Ala5^*), resulting in the loss of >95% of all hemocytes (Fig. 2A). In these embryos, levels of the basal lamina compound perlecan are strongly reduced, showing a graded distribution along the anterior-posterior axis (Fig. 2B, gray arrows). The near loss of the basal lamina (or its integrity) results in a failure of nerve chord condensation that normally occurs from 13-17 h AEL (Fig. 2C) (Martinek et al., 2008; Olofsson and Page, 2005). Remarkably, this reduction of the basal lamina has no effect on SPG migration or polarity (Fig. 2D), but causes severe defects in SPG morphology. As revealed by Dg labeling, the SPG are smaller compared to age-matched controls and fail to form a contiguous epithelium (Fig. 2D). These defects are worse in the posterior regions of the CNS, indicating that glial growth is correlated with the protein levels of basal lamina components. As a result, a BBB never forms, as shown by the strong penetration of a charged fluorescent dye (10 kD dextran) into the nerve cord of 22 h-old embryos, i.e. at a time when dye is completely excluded in wild type (WT) (Fig. 2C). These data demonstrate that SPG growth is very sensitive to (partial) depletion of the basal lamina, while SPG migration and polarity are not.

### Misregulation of G protein signaling leads to glial growth defects

In a previous study, we had identified a putative GPCR signaling pathway (called the ‘Moody pathway’ for short) that is required for BBB formation (Schwabe et al., 2005) and the insulation defects observed in pathway mutants are attributable to maldistribution of SJs along the cell perimeter. However, the study focused on late stages of BBB development, leaving open the question when and in which cells the defects first arise. We therefore examined how the different stages of MET are affected by misregulation of the pathway.

The pathway consists of the orphan GPCR Moody, the regulator of G protein signaling (RGS) Loco, as well as two heterotrimeric G proteins, Gαi and Gαo, that bind a common Gβγ subunit (Gβ13F, γ1); the main effector signaling is mediated by Gαo and Gβγ. While both Moody and the heterotrimeric G proteins are positive regulators in the pathway, both structural and genetic evidence suggests that Loco acts as a negative regulator, by promoting inactivation of Gα signaling via its RGS domain (Schwabe et al., 2005; Siderovski and Willard, 2005). Supporting this notion, we find that the BBB defect of *loco* mutants is completely rescued by expression of a truncated Loco protein containing only the RGS domain (Fig. S2). Thus, to examine loss of pathway activity, we use *moody* zygotic mutants or glial overexpression of constitutively inactive Gαo^GDP^. To examine pathway overactivity, we use *loco* zygotic mutants (*loco^Z^*) or constitutively active Gαo^GTP^ (Schwabe et al., 2005). Additional removal of *loco*’s strong maternal component (*loco^MZ^*) leads to more severe insulation defects (Fig. S2), but with the complication that the embryos show mild neurogenesis defects resulting in the occasional loss of individual SPG cells (Yu et al., 2005).

The first stage of BBB formation is the migration of SPG onto the surface of the nerve cord. The timing of this migration is unaffected in all Moody pathway mutants (Table S1).

To examine whether the Moody pathway impacts glial growth, we performed a time-lapse analysis of SPG behavior between 11 and 13 h by tracing individual cell contours to measure various metrics to quantify cell shape and growth (see Materials and Methods).

**Fig. 3.**
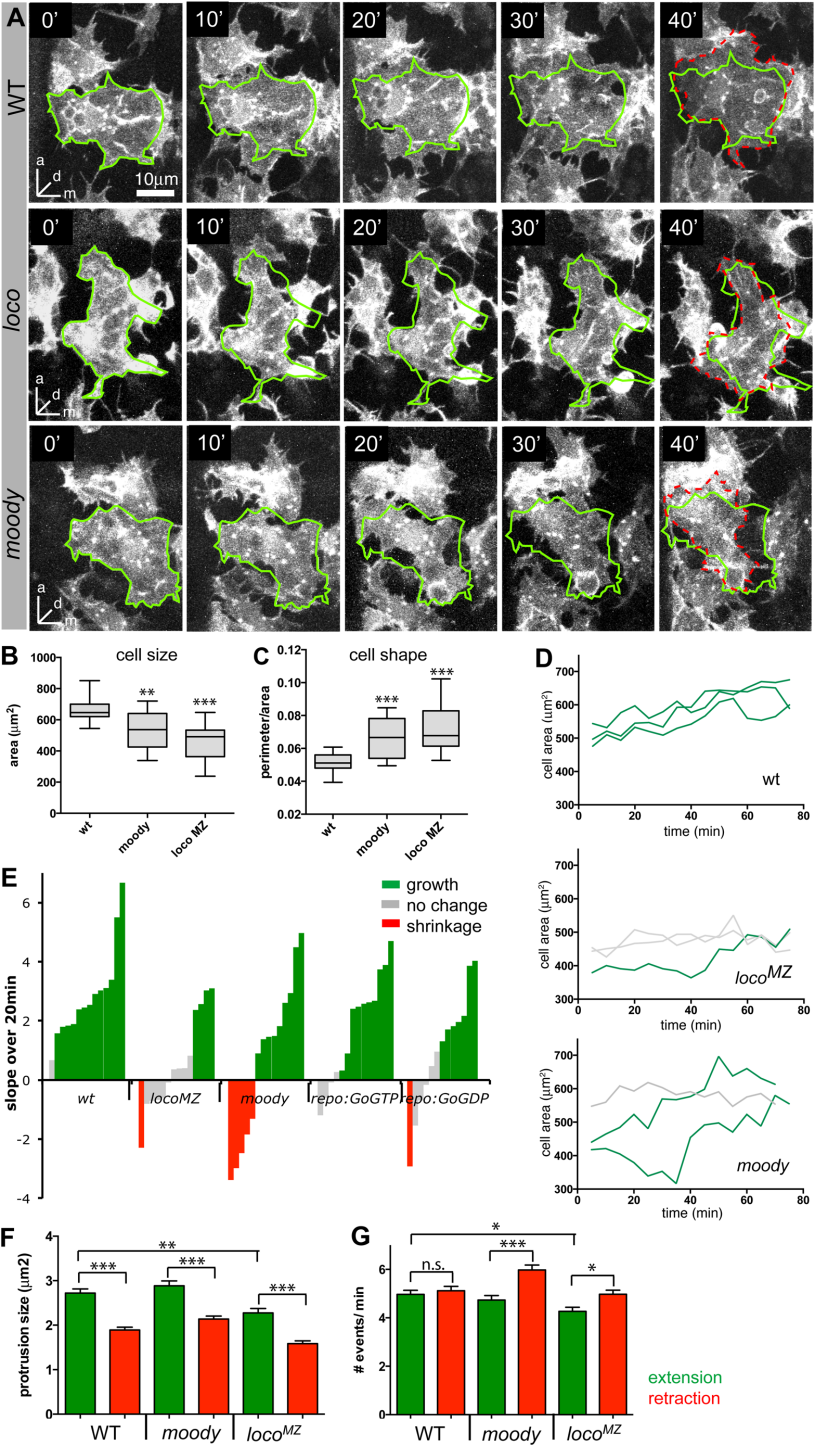
**Moody signaling regulates SPG growth.** (A) Movie stills of representative SPG in WT, *loco^MZ^* and *moody* mutants. *loco* and *moody* mutant SPG often fail to stabilize protrusions and show slower overall growth. Glia are labeled with *repo>GFPmoesin,**>**gapGFP*; ventral views; stacks of 6 µm depth. The green outline represents the cell at 0 min and the hatched red line the cell outline at 40 min. (B,C) Box plots showing median, interquartile range, and total range; *n*=16-19 per genotype. (B) *moody* and *loco* mutant SPG are significantly smaller and more variable in size than WT SPG. (C) Both *loco* and *moody* mutants also have less compact and more variable cell shape, measured as the ratio of cell perimeter/area; other metrics of compactness give similar results. (D) Growth curves of three representative cells in WT, *loco^MZ^* and *moody* embryos over 75 min. (E) Cell growth in WT and *moody* pathway mutants, as represented by the slope of the fitted linear regression line on cell growth over 20 min (*n*=13-14 per genotype); a positive slope indicates cell growth, while a negative slope indicates shrinkage. Each bar represents one cell. Significant growth is marked in green, no change in gray and significant shrinkage in red. (F) Average number of extensions (green) and retractions (red) per minute in WT and moody pathway mutants. Both *loco^MZ^* and *moody* show an increase in the number of retractions, while *loco^MZ^* also shows significant reduction in the number of extensions. (G) Average size of extensions or retractions in the different genotypes. Extensions are larger than retractions in all genotypes, but *loco^MZ^* mutants also show reduced extension sizes. (B, C, F, G) Data were analyzed by *t*-test adjusted for the number of comparisons made, *P<0.05, **P<0.01, ***P<0.001.

WT SPG have a compact shape and uniform size (Fig. 3A-C), with 13 out of 14 measured cells showing significant and synchronized growth over periods of both 20 min and 75 min (Fig. 3D,E; Movie 1). *Moody* mutant SPG show less compact and more variable cell shapes (Fig. 3A-C), and their size is smaller and more variable than in WT (Fig. 3B,C). The SPG in *moody* mutants also show slightly retarded and much more variable growth behavior: while the majority of cells do grow, some (5 out of 14) significantly decrease in size over a 20 min time interval (Fig. 3D,E; Movie 2).

Since our time lapse analysis focuses on short time windows, we used the stronger maternal and zygotic *loco* mutants (*loco^MZ^*) to assess the effects of pathway overactivity, but selected embryos with normal numbers of SPG and PNG. Similar to *moody* mutants, *loco^MZ^* mutant SPG are smaller than in WT, and show highly irregular and variable cell shapes (Fig. 3A-C) as well as retarded growth (Fig. 3D,E; Movie 3). Over 20 min, and even over a period of 75 min, only a minority of *loco^MZ^* cells grow, while some shrink and the majority show no significant change in size. Comparable, albeit weaker, defects are observed when the *moody* pathway is misregulated by glial overexpression of either Gαo^GTP^ or Gαo^GDP^ (Fig. 3E). These weaker phenotypes are likely due to low levels of transgene expression, as the *repoGal4* driver becomes active only 2 h prior to the time-lapse analysis.

Similar to the events at the leading edge of migrating cells, spreading cells continuously generate extensions and retractions around their circumference. Some of the extensions are stabilized through adhesive interaction with the substrate, leading to a net increase in cell size. To better understand the nature of the growth defects we observe in *moody* pathway mutants, we measured both filopodial and lamellipodial extensions and retractions per cell per minute, as well as their average length and sizes. We found no differences in filopodial length, number or lifetime in the GPCR mutants (data not shown). Focusing on lamelliopodia, in WT animals protrusions are larger on average than retractions (Fig. 3F), although both occur with equal frequency (Fig. 3G). This suggests that when a protrusion forms and extends, part of it stabilizes and part of it retracts. Due to stabilization of the protrusion, WT SPG continuously increase in size over time. Also, in *moody* and *loco^MZ^* mutants, extensions are larger than retractions, suggesting that the initial stabilization does occur equally well. However, in both mutants the number of retractions significantly exceeds the number of extensions, suggesting that cell substrate contacts are not stabilized as well over time. This is also reflected in the change of cell contours over time (Fig. 3A). In WT, almost all areas covered at 0 min are still covered after 40 min, and additional areas are covered by new growth. In *moody* and *loco^MZ^* mutants, by contrast, large areas covered at 0 min are no longer covered after 40 min. Finally, extensions are significantly smaller in *loco^MZ^* mutants, consistent with their retarded overall growth.

Thus, in sum, both pathway under- and overactivity lead to a reduction in SPG cell size, compactness and growth, and to an increase in variability for all these parameters. Looking at growth behavior in greater detail, we find that both *moody* and *loco* destabilize cell substrate contacts. *moody* shows greater variability in growth, while *loco* reduces protrusion size and frequency, leading to more retarded growth.

### Insulation defects in GPCR signaling mutants are a consequence of growth defects

**Fig. 4.**
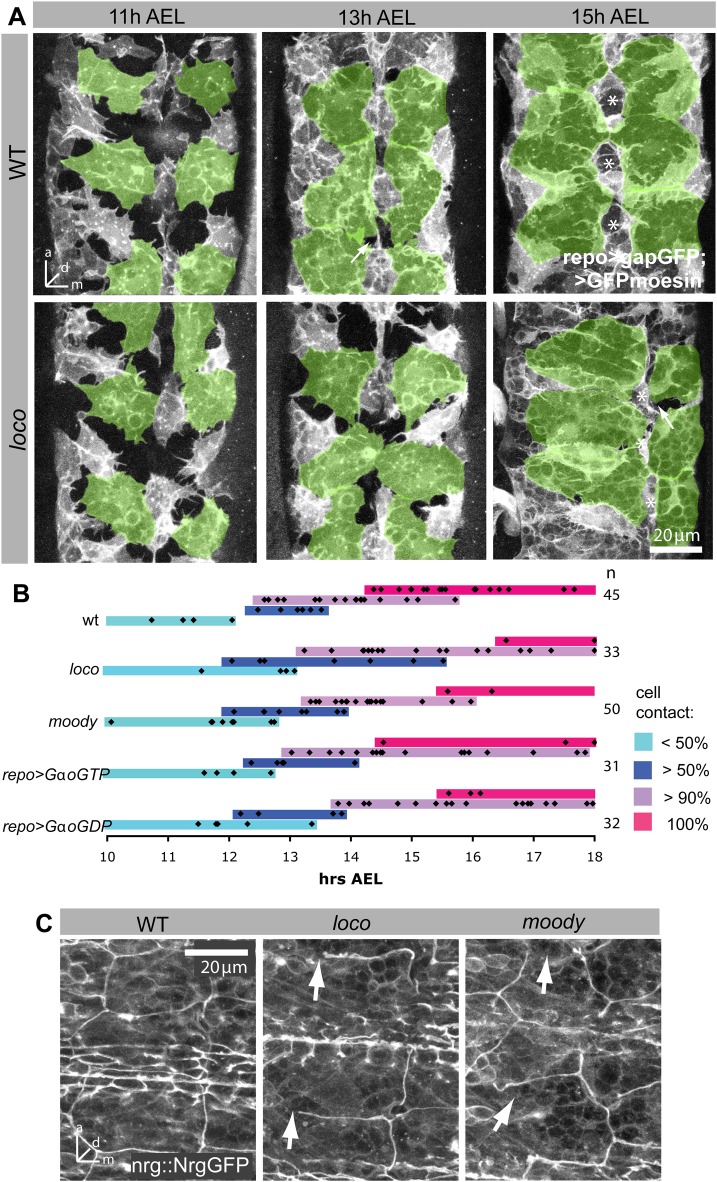
**Moody signaling regulates timing and coordination of SPG epithelium formation.** (A) Morphology of SPG at different time points in WT and *loco* zygotic mutant embryos. In *loco* mutants, SPG shape and size is variable and epithelial closure is delayed. SPG are labeled by *repo>GFPMoesin, >gapGFP* and ventral SPG are highlighted in green; live images, stacks of 8-14 µm. Stars label midline channels, which may be PNG that surround a midline channel. (B) Graph summarizing epithelial development in different Moody pathway mutants. Removal of *moody* or *loco*, and glial overexpression of *Gαo^GTP^* or *Gαo^GDP^* all result in a delay of epithelial development. Black diamonds represent individual embryos. Colored bars indicate stage of MET: light blue - SPG form <50% cell contact with each other; dark blue - SPG form >50% cell contacts, but large holes remain visible; purple - only few small gaps are visible; magenta - the epithelium has closed completely. (C) The timing of SJ formation in *moody* and *loco* mutants is similar to WT. However, several large gaps are visible in both mutants (arrows). Embryos are 16 h AEL. SJs are labeled by Nrg::GFP; ventral view of CNS; stacks of 11 µm.

Next we wanted to see how these defects in glial growth affect epithelium formation by SPG. Using the same markers for SPG and imaging live embryos at various stages of development, we found that epithelial closure in all GPCR mutants is significantly delayed by at least 1 h (Fig. 4A,B). Only repo>GaoGTP overexpressing embryos appear to have no delay in epithelial formation, which is in line with weaker growth defects observed (Fig. 3E).

Yet despite the delay in epithelium development, SJ formation (as labeled by Nrg::GFP) begins at the normal time in *loco* and *moody* mutants (Fig. 4C). In WT epithelial closure occurs at 14.5-15.5 h, while the beginning of SJ formation occurs at 15.5-16.5 h. The two processes overlap in the GPCR pathway mutants. When we examine SJ distribution at 16 h, junctions are found uniformly along the entire cell circumference in WT, but many gaps appear in the junction belt of *loco* and *moody* mutants (Fig. 4C), likely due to the lack of completion of cell contact formation between neighboring glia. Our data thus indicate that the Moody pathway is required for epithelial morphogenesis already prior to the formation of the SJ belt, but does not directly impact the timing of SJ formation.

### Septate junctions are critical for polarity of SPG

**Fig. 5.**
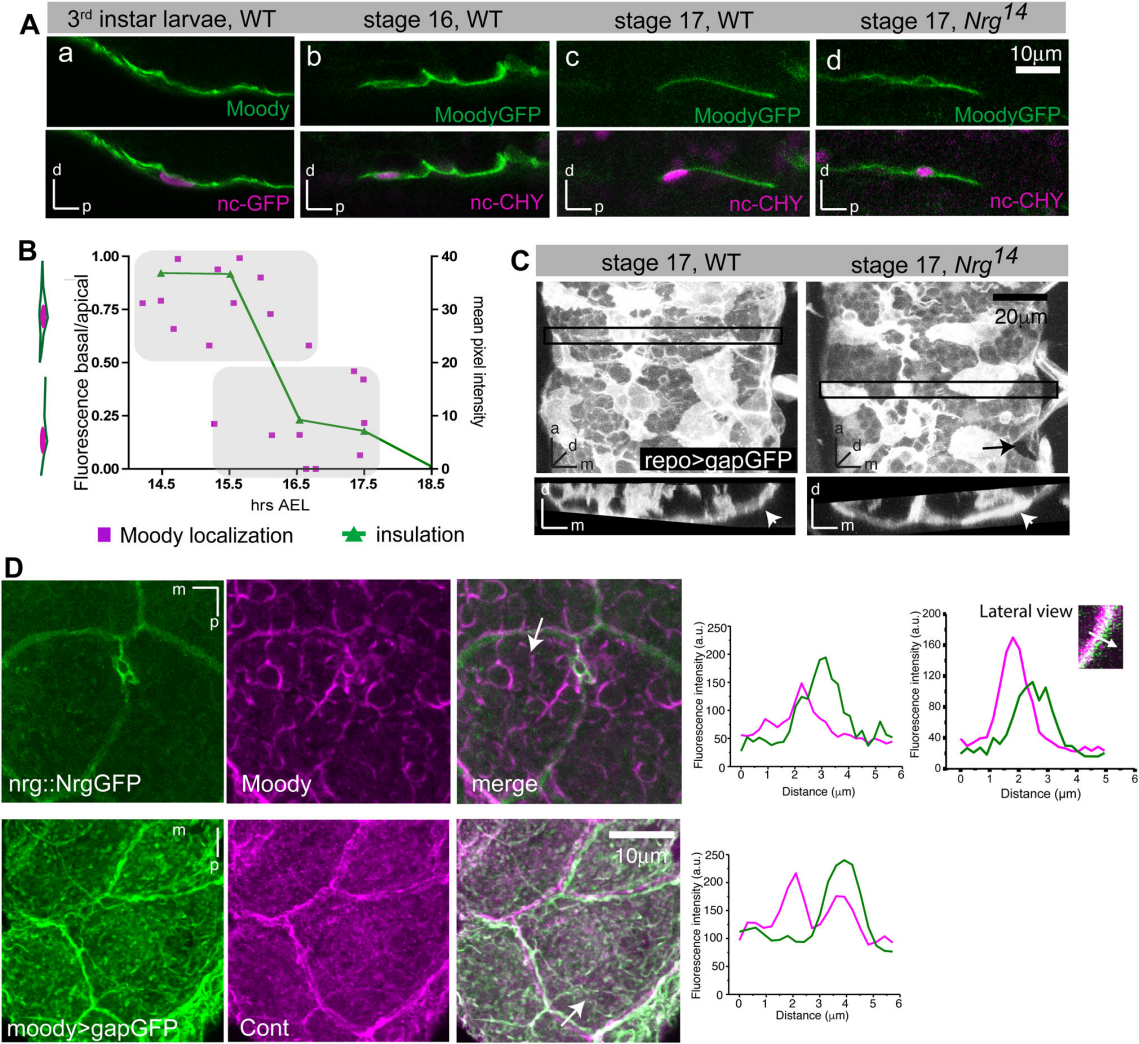
**Fence function of SJs is required for establishment of polarized membrane compartments in SPG.** (Aa) Immunohistochemistry against Moody and *moody>ncGFP*. Moody is enriched on the apical (i.e. nervous-system facing) side of SPG in third instar larva. (Ab-d) Live imaging of GFP-tagged Moody using *MZ1251>moodyGFP, nuclearCherry*. Moody is not polarized in stage 16 embryos, but localizes exclusively to the apical surface by stage 17. (Ad) In *Nrg^14^* mutants, Moody fails to polarize. Lateral views of the CNS/hemolymph border with the CNS facing up in all images. (B) Quantification of MoodyGFP localization in WT. Polarization of MoodyGFP (magenta), computed as basal/apical ratio of fluorescence intensities, coincides with CNS insulation (green). Insulation was quantified by measuring the levels of fluorescent Dextran-TR diffusion into the CNS. (C) In *Nrg^14^* mutants, the SPG epithelium forms largely normally (arrowheads), although small gaps in the epithelium remain visible (arrow). 17 h old embryos; glia are labeled by *repo>gapGFP*; top panels, ventral view; bottom panels, orthogonal view of areas indicated by black box; stack of 15 µm. (D) Both Moody and gapGFP are observed immediately adjacent to but not coincident with SJ components (Nrg::GFP or Contactin). Immunohistochemistry; ventral view of CNS; stacks of 10 µm; graphs show intensity profiles along the line marked by an arrow in the merged panels.

Once the SPG epithelium has formed, cells establish polarized membrane compartments. The ABC transporter Mdr65 is restricted to the hemolymph facing basal membrane (Mayer et al., 2009); by contrast, the GPCR Moody is restricted to the apical membrane, which faces the nervous system (Fig. 5Aa) (Mayer et al., 2009).

To follow the distribution of Moody protein throughout epithelial development during embryogenesis, we expressed a GFP-tagged version of the protein at moderately elevated levels using the *MZ1251-Gal4* driver (Ito et al., 1995); the endogenous protein levels are too low to perform fluorescent immunohistochemistry. Intriguingly, we find that prior to epithelial closure, Moody localizes uniformly to all membrane compartments (Fig. 5Ab). Coincident with CNS insulation, however, Moody distribution becomes specifically localized to the apical membrane compartment (Fig. 5Ac,B), suggesting that the formation of lateral SJs is necessary for generating polarized Moody localization. To test this idea directly, we examined embryos mutant for the SJ components *Nrg* and *Nrx–IV*, in which SJs do not form. In both mutants, MoodyGFP remains ubiquitously localized until late embryogenesis (Fig. 5Ad, data not shown), demonstrating that SJs are necessary for the establishment of distinct membrane compartments within the SPG. Notably, the lack of Moody polarization is not due to a failure of epithelial closure, as the glial epithelium forms largely normally in the absence of SJs (Fig. 5C). This finding indicates that SJs play an essential role in blocking diffusion not only in the paracellular space but also within the plasma membrane.

Support for this notion comes from double-labeling experiments: in SPG of third instar larvae, co-labeling of endogenous Moody protein and the SJ marker Nrg::GFP shows that Moody is adjacent to, but not overlapping with, the lateral Nrg::GFP, suggesting that it is indeed excluded from the lateral membrane compartment (Fig. 5D). We observe a similar lateral exclusion of the membrane-bound gapGFP (Fig. 5D), suggesting that SJs form a diffusion barrier within the membrane, which would effectively prevent intermixing of proteins of the apical and basal membrane compartments. A similar fence function has been described for the vertebrate SJs found at the paranodal junction of myelinated axons, where they restrict diffusion of potassium channels within axonal compartments (Bhat et al., 2001).

## DISCUSSION

Our study of *Drosophila* BBB development represents the first dynamic *in vivo* study of MET and secondary epithelium formation. Our data shed particular light on the roles of the basal lamina and of the insulating SJs, as well as on the function of GPCR signaling in this important morphogenetic process.

Once SPG reach the CNS surface, contact with the basal lamina is essential for the extensive growth of the SPG during epithelium formation. Previous *in vitro* studies have shown that adhesion to basal lamina components is necessary for cell spreading and proliferation (Folkman and Moscona, 1978; Huang and Ingber, 1999), however our study is the first to demonstrate *in vivo* that attachment to the basal lamina is essential for non-proliferative cell growth and ensheathment. Attachment to the ECM occurs primarily through focal adhesions and integrins (Bökel and Brown, 2002), which in turn can activate MAPK signaling, triggering cell proliferation and growth (Boudreau and Jones, 1999). In addition, adhesion to the ECM has been shown to provide traction, which facilitates cell spreading (Huang and Ingber, 1999). Contact to the ECM may thus provide the SPG with both growth signals and attachment sites. Being highly expressed on the basal lamina facing side of SPG, Dg is an excellent candidate for mediating ECM attachment. However, zygotic mutants of Dg show no BBB defects (data not shown) and germline clones could not be analyzed due to Dg's role in oogenesis (Deng et al., 2003).

**Fig. 6.**
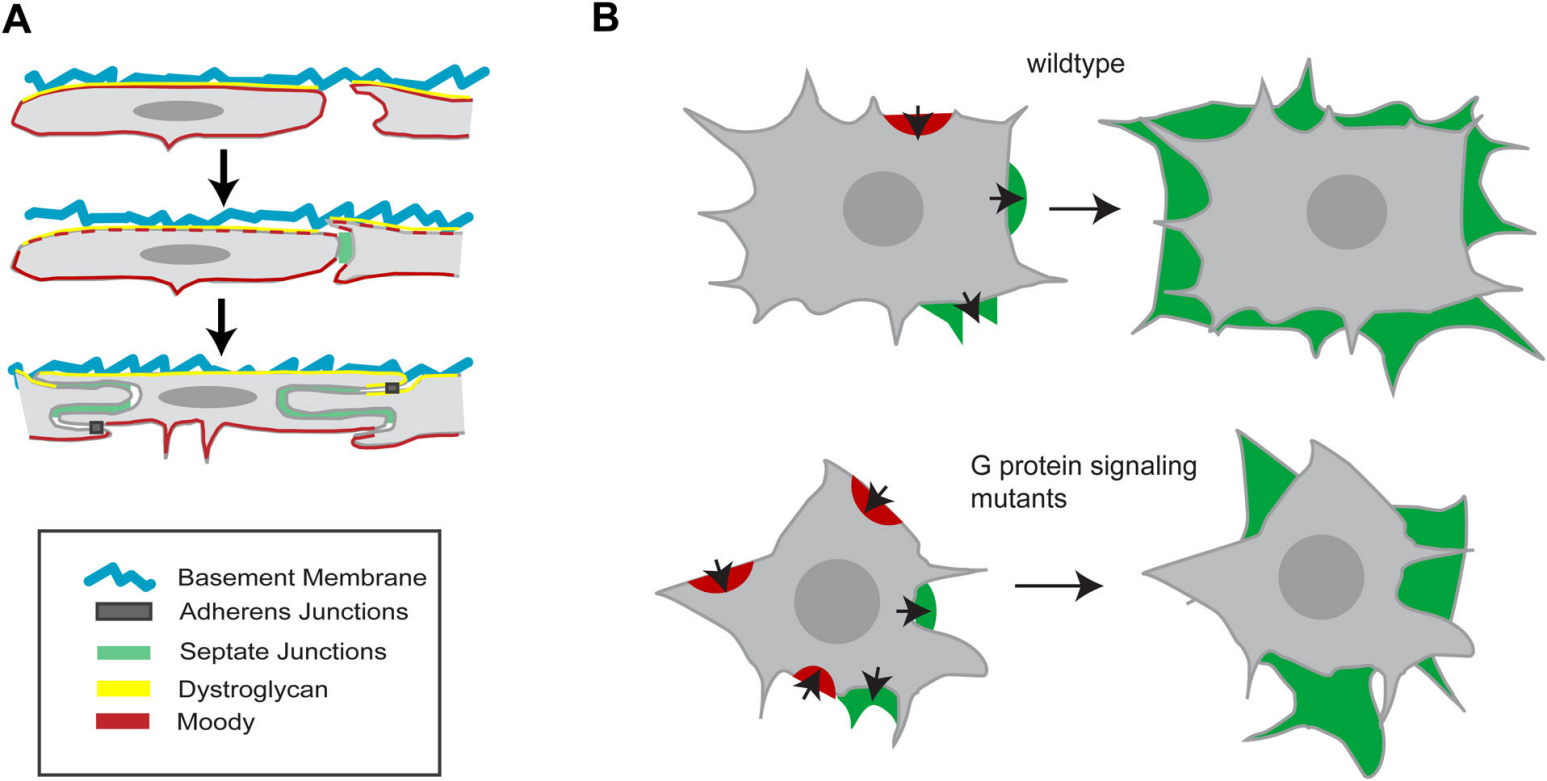
**Model summarizing SPG MET and BBB formation.** (A) Formation of polarized domains in SPG. In contrast to columnar primary epithelia, SPG form a squamous epithelium and lack classical apical polarity complexes. Dystroglycan (Dg) shows polarized distribution in the glia prior to epithelial formation, suggesting that initial apical-basal polarity is established by contact to the basal lamina. Moody polarity depends on the presence of SJs. (B) SPG growth is regulated by GPCR pathway activity and there is an increase in the number of retractions in GPCR pathway mutants.

Beyond supporting SPG growth, contact with the basal lamina likely provides an important cue for polarizing the cells, as judged by their strong enrichment of Dg at the basal lamina facing (basal) membrane compartment (Fig. 6A). Previous studies have shown that Dg and its ligand Pcan are required for the establishment of polarity in follicle cells (Deng et al., 2003; Schneider et al., 2006). However, when we deplete the basal lamina and thus its ligand Pcan, Dg is still expressed and polarized in the SPG, suggesting that glial polarity can be supported by the residual basal lamina or that additional polarizing signals exist.

Once SJs have formed, the GPCR Moody and the Mdr65 transporter are asymmetrically distributed within the SPG, further demonstrating that these cells possess distinct apical and basal membrane compartments. We could show that this polarized distribution is coincident with and dependent on the presence of SJs, demonstrating for the first time that SJs serve a function in cell polarity (Fig. 6A). By acting as a fence and preventing diffusion of membrane proteins across the lateral compartment, the SJs maintain asymmetric protein distributions, which could result from polarized exocytosis or endocytosis. Intriguingly, we have in a separate study identified PKA as a crucial antagonistic effector of Moody signaling (X. Li, R. Fetter, T.S., C. Jung, H. Steller, U.G., in preparation). PKA has been shown to regulate polarized exocytosis at the *trans*-Golgi network in different types of epithelia (Wojtal et al., 2008). Apical-basal polarity plays an important morphogenetic role in the continued growth of the SPG epithelium during larval stages (X. Li, R. Fetter, T.S., C. Jung, H. Steller, U.G., in prep.) and in the function of the BBB (Mayer et al., 2009).

Signaling by the GPCR Moody plays a critical role both in regulating the growth of individual SPG and in synchronizing this process across the entire SPG cell population. In Moody pathway mutants, glial growth behavior is more erratic, and more variable between cells. This increased variability of glial cell shape, size, and growth causes a significant delay of epithelial closure of up to 1.5 h. This delay is not caused by an earlier delay in glial migration or by a delay in SJ formation.

The detailed dynamic analysis reveals that, in *moody* and *loco* mutants, the spatio-temporal coordination of cell spreading is impaired. Spreading cells (Xiong et al., 2010; Ryan et al., 2012), like other motile cells, show fluctuating exploratory motions of the leading edge visible as cycles of protrusion and retraction. This complex process can be broken down into discrete steps: actin protrusion of the leading edge, adhesion to the ECM, and myosin-driven contraction against adhesions. Our time-lapse recordings indicate that Moody signaling has its most pronounced effect on the stabilization of protrusions, as evidenced by an increase in the ratio of retractions to extensions, and the marked shift of cell contours over time (Fig. 6B). The destabilization of protrusions might be due to weaker integrin-mediated interaction of focal adhesions with the ECM, but also due to impaired stress-mediated maturation of focal adhesions (Gardel et al., 2010). The fact that both under- and overactivity of the Moody pathway impair protrusion stabilization may be due to the feedback between actin-myosin and focal adhesion, which also causes the well-known biphasic response of migration speed to adhesion strength of migrating cells (Gupton and Waterman-Storer, 2006). While the loss of *moody* has no significant effects on the other parameters we measured, the loss of *loco* also reduces the frequency and size of protrusions, suggesting that actin polymerization may be specifically affected by increased GPCR signaling activity. Cumulatively, these impairments in protrusion/retraction behavior lead to retarded, non-isometric growth of SPG and to the irregular cell shapes observed in *moody* and *loco* mutants.

Interestingly, we have recently identified PKA, Rho1 and MLCK as important downstream effectors of Moody signaling (X. Li, R. Fetter, T.S., C. Jung, H. Steller, U.G., in prep). All three factors are well known to control actin-myosin contraction – Rho1 and MLCK as positive regulators and PKA as a negative regulator. More recently, Rho1 activity has been shown to also drive actin polymerization at the leading edge (Machacek et al., 2009), and a PKA-RhoGDI-Rho1 regulators feedback loop has been suggested to act as a pacemaker of protrusion-retraction cycles (Tkachenko et al., 2011).

The role of Moody pathway signaling in directed and well-coordinated cell growth is strikingly similar to the function of trimeric G protein signaling in other contexts. In *Dictyostelium*, G protein signaling is essential for directed cell migration. When all G protein signaling is abolished, cells are still mobile and actively generate protrusions, however these protrusions form in random directions (Sasaki et al., 2007), with the result that the cells lose their directionality. During gastrulation in *Drosophila*, signaling by the Gα12 ortholog Concertina and the putative GPCR ligand Folded gastrulation synchronizes apical actin-myosin constrictions of mesodermal precursor cells, and thereby effects their concerted invagination (Parks and Wieschaus, 1991; Costa et al., 1994). Thus, a major role of G protein signaling during development may be to modulate basic cellular behaviors such as cell growth, protrusion, or contraction, and reduce variability within cells and between neighboring cells with the goal of generating uniform patterns and behaviors.

Synchronized growth behavior of SPG is not only important for rapid epithelial closure but, ultimately, also for generating an evenly sealed BBB. All our evidence supports the notion that the defects in SJ organization that are responsible for the BBB failure are a secondary consequence of the morphogenetic function of the GPCR pathway. Cell contacts precede and are necessary for SJ formation, and the growth of cell contacts and SJ accumulation are strongly correlated. Delayed and more erratic cell-cell contact formation, as is the case in Moody pathway mutants, is likely to result in uneven circumferential distribution of SJ material later on; conversely, the timing of SJ formation per se is not affected by the pathway, arguing against a direct effect. Since the insulating function of SJs depends on their length, a decrease in the length in some local areas will result in insulation defects. Moreover, since SJs are known to form very static complexes (Oshima and Fehon, 2011), any irregularity in SJ distribution may be retained for long periods of time.

Although under- and overactivity of the Moody pathway lead to globally similar outcomes, impaired epithelium formation and failure of BBB insulation, our data point to subtly different subcellular effects of the two types of pathway modulation. During MET, *loco* mutants (which we confirm indeed induce pathway overactivity) show predominantly retarded growth, presumably as a result of curtailed protrusive activity, while *moody* mutants show severe fluctuation and variability in growth. It will be very interesting to investigate these distinct outcomes of Moody pathway misregulation in greater detail.

## MATERIALS AND METHODS

### Fly strains and constructs

The following fly strains were obtained from published sources: *moody^Δ17^* (R. Bainton, Bainton et al., 2005); *MZ1251*, *loco^Δ13^* (C. Klämbt; Granderath et al., 1999); *loco^P283^*, *UAS-Gαi^GDP^* (W. Chia; Yu et al., 2003); *Nrg^14^* (M. Hortsch, Godenschwege et al., 2006); *Nrg^G305^* (Nrg::GFP) (L. Cooley, Quiñones-Coello et al., 2007); *Nrx-IV^4025^* (M. Bhat; Baumgartner et al., 1996); *repo-Gal4* (V. Auld, Sepp and Auld, 1999); *moody-Gal4* (Schwabe et al., 2005); *UAS-GFPmoesin* (D. Kiehart, Dutta et al., 2002); *UAS-Gαo^GTP^* and *UAS-Gαo^GDP^* (A. Tomlinson, Katanaev et al., 2006); *UAS-ncGFP*, *UAS-gapGFP*, *UAS-myrRFP* [Bloomington *Drosophila* Stock Center; A. Chiba (Department of Biology, University of Miami, 1301 Memorial Drive, Coral Gables, FL 33146, USA) personal communication to Flybase], *UAS-hid^Ala5^* (H. Steller, Bergmann et al., 1998); *UAS-locoRNAi* (V4291, VDRC; Dietzl et al., 2007); *UAS-moodyRNAi* (R. Bainton, Bainton et al., 2005); *croquemort-Gal4*,*UAS-GFP* (gift from N. Franc, Cuttell et al., 2008). *UAS-nucCherry* (*ncCHY*; mCherry by R. Tsien, Shaner, et al., 2004) was generated by removal of the mCherry stop codon and cloning it in place of ECFP into pECFP-Nc (Clontech, Takara Bio USA, Inc., CA, USA). *UAS-CHYMoesin* was generated by substituting GFP of GFPMoesin with mCherry using the same restriction sites as D. Kiehart (Edwards et al., 1997). *UAS-moodyα/βGFP* was generated by in-frame fusion of EGFP to the C-terminus of the α and β splice forms of Moody. Expression of either Moodyα or MoodyβEGFP in glia using *repoGal4* rescued adult lethality of *moody^C17^* mutants. To balance most of our mutants we used *FM7c-KrGal4>UASGFP*, *CyO- KrGal4>UASGFP* and *TM6B-KrGal4>UASGFP* balancer chromosomes (Bloomington *Drosophila* Stock Center). Maternal and zygotic mutants were generated by crossing zygotic mutant females that survived to adulthood with heterozygous males. Subcellular localization of both splice forms is identical at all stages of BBB development and images shown in Fig. 3 are from *UAS-MoodyβGFP*. All constructs from above were cloned into pUAST (Brand and Perrimon, 1993). Mutant and transgenic lines were genotyped using fluorescently labeled balancers. Late stage 17 *Nrg* and *Nrx-IV* mutants were identified by the lack of tracheal air-filling, and by dye penetration through the epidermis and into the ventral nerve chord. For all live experiments, embryos and larvae were raised at 25°C.

### Immunohistochemistry

Immunohistochemistry followed standard procedures using rabbit anti-Repo (1:100, Gaul lab, Gene Center, Munich, Germany), mouse anti-Repo (1:5, DSHB, University of Iowa, Department of Biology, IA, USA), sheep anti-GFP (1:100, Biogenesis, Planegg, Germany), mouse anti-GFP (1:250, Molecular Probes, Thermo Fisher Scientific, MA, USA), guinea pig anti-Contactin (1:2000, M. Bhat, Physiology, University of Texas School of Medicine, TX, USA), rabbit anti-RFP (1:200, US Biological, MA, USA), rabbit anti-Dystroglycan (1:500, H. Ruohola-Baker, Department of Biochemistry, University of Washington, Seattle, WA, USA), rabbit anti-Lamininγ (1:100, DSHB), rabbit anti-Perlecan (1:500, S. Baumgartner, Developmental Biology, Lund University, Lund, Sweden). Fluorescent secondary antibodies were coupled to Cy3 (1:200, Jackson ImmunoResearch) or Alexa Fluor 488 (1:200, Invitrogen/Molecular Probes). Rat anti-Moody β was generated according to Bainton et al. (2005). Specificity of immune sera was determined by immunohistochemistry in third instar larvae (1:500). In WT, Moody strongly labels SPG, while *moody^C17^* mutant larvae show no signal (data not shown).

### Live imaging and data analysis

Live imaging was carried out as follows: dechorionated embryos of varying stages were mounted under halocarbon oil. Embryos older than 16 h AEL were injected with 100 mM potassium cyanide (Sigma, 2-3% of egg volume) to subdue their movement, and imaged 30 to maximal 60 min after injection. Dissected third instar cephalic complexes were mounted in saline and imaged directly. All confocal images were acquired using a Zeiss LSM 510 system using standard settings (pinhole 1, z-section thickness 0.5 µm). Images were analyzed using Zeiss LSM 510 software. Glial growth in Fig. 1C was measured by live imaging of SPG every 30 min. To measure both surface area and volume of SPG *in vivo*, we cropped individual SPG from surrounding Repo-positive glia and built a 3D cell model by iso-surfacing with appropriate thresholds in Imaris 4.0 (Bitplane). We then averaged volume and surface area of all SPG modeled in this fashion to obtain growth curves.

Time-lapse microscopy was carried out at 20°C on embryos of about 11 h AEL using an inverted Zeiss LSM 510 confocal microscope. To increase signal strength, the pinhole was opened to 1.3 (z-section thickness 0.6 µm). Z-stacks of 12 sections were acquired once per minute. To adjust for focus-drift, which is mainly caused by rotation of the embryo, the Z-stack coordinates were adjusted at various time points. Between 5 and 7 movies were captured per genotype, each of 80-110 min duration. Quantitative image analysis was performed using ImageJ 1.37v (NIH); cell outlines of individual SPG were traced manually, and parameters such as cell area and perimeter extracted. Glial growth was measured by performing a linear regression analysis on cell area over time. The slope of the line represents the growth rate, while the correlation coefficient R allows us to distinguish significant growth (R approaches 1) from shrinkage (R approaches −1) and no growth/change (R approaches 0). To measure the frequency and size of extensions and retractions, a cell's outline was traced and this trace was transferred to t+1 min. All areas protruding over this outline were traced and measured as individual extensions and all areas receding from the outline were traced as individual retractions. 20 time points were analyzed for each cell. Statistical analyses were performed using GraphPad Prism. For pair-wise comparisons, Student's *t*-test was performed; for comparing multiple groups, we performed one-way ANOVA with Dunnett or Student–Newman Keuls post hoc test.

To measure SJ width in larvae, we used the Imaris Software package to perform 2D segmentation on maximum intensity projections of 3D confocal stacks of Nrg-labeled nervous systems. To obtain the mean width of the SJs in an animal, we split the segmentation patterns into multiple segments of 3–4 µm in length, then extracted and averaged the ellipsoid axis lengths along their perpendicular axis.

### Staging of embryos and dye injections

To precisely stage live embryos, we used standard morphological markers, such as midgut development, and combined this with a novel approach which uses the condensation of the ventral nerve chord along its anterior-posterior axis as a reliable measure of age in embryos between 11 and 18 h AEL. We measured condensation in WT embryos, plotted the mean segment width against time and performed a linear regression analysis. The trend line is used as a reference to calculate the age of embryos (Fig. S3). Since CNS condensation is mildly impaired in *loco*, *Nrg* and *repo>Gαo^GTP^* and *repo>Gαo^GDP^* mutants, separate reference trend lines were established for these genotypes. Embryonic dye injections were performed as described in (Schwabe et al., 2005).

### Transmission electron microscopy

Embryos were processed by high pressure freezing in 20% BSA, freeze-substituted with 2% OsO4, 1% glutaraldehyde and 0.2% uranyl acetate in acetone (90%), dH_2_O (5%), methanol (5%) over 3 days (−90°C to 0°C), washed with acetone on ice, replaced with ethanol, infiltrated and embedded in Spurr's resin, sectioned at 80 nm and stained with 2% uranyl acetate and 1% lead citrate for 5 min each. Sections were examined with a Fei Tecnai G2 Spirit BioTwin Transmission Electron Microscope with a Gatan 4K×4K digital camera. For conventional TEM, third instar larvae were dissected and fixed in 4% glutaraldehyde, after which they were processed as described in Auld et al. (1995).
